# Applying the WHO-ICRC BEC course to train emergency and inpatient healthcare workers in Sierra Leone early in the COVID-19 outbreak

**DOI:** 10.1186/s12913-022-07556-8

**Published:** 2022-02-14

**Authors:** Paul D Sonenthal, Chiyembekezo Kachimanga, Doris Komba, Moses Bangura, Nicholas Ludmer, Marta Lado, Marta Patino, Rachel B Gerrard, Matthew J Vandy, Regan H Marsh, Joia Mukherjee, Shada A Rouhani

**Affiliations:** 1 Brigham and Womens Hospital, Division of Pulmonary and Critical Care Medicine, 75 Francis Street Massachusetts, Boston, MA 02115 USA; 2 Harvard Medical School, Boston, MA USA; 3 Partners In Health, Boston, MA USA; 4 Partners In Health-Sierra Leone, Kono, Sierra Leone; 5 Ministry of Health and Sanitation, Freetown, Sierra Leone; 6 University of Chicago, Section of Emergency Medicine, Chicago, IL USA; 7 Brigham and Womens Hospital, Department of Emergency Medicine, Boston, MA USA

**Keywords:** Emergency and critical care, COVID-19, Basic emergency care course, Capacity building, Training

## Abstract

**Background:**

Treating critical illness in resource-limited settings during disease outbreaks is feasible and can save lives. Lack of trained healthcare workers is a major barrier to COVID-19 response. There is an urgent need to train healthcare workers to manage COVID-19. The World Health Organization and International Committee of the Red Cross’s Basic Emergency Care course could provide a framework to cross-train personnel for COVID-19 care while strengthening essential health services.

**Methods:**

We conducted a prospective cohort study evaluating the Basic Emergency Care course for healthcare workers from emergency and inpatient units at two hospitals in Sierra Leone, a low-income country in West Africa. Baseline, post-course, and six month assessments of knowledge and confidence were completed. Questions on COVID-19 were added at six months. We compared change from baseline in knowledge scores and proportions of participants “very comfortable” with course skills using paired Student’s t-tests and McNemar’s exact tests, respectively.

**Results:**

We enrolled 32 participants of whom 31 completed pre- and post-course assessments. Six month knowledge and confidence assessments were completed by 15 and 20 participants, respectively. Mean knowledge score post-course was 85% (95% CI: 82% to 88%), which was increased from baseline (53%, 48% to 57%, *p*-value < 0.001). There was sustained improvement from baseline at six months (73%, 67% to 80%, *p*-value 0.001). The percentage of participants who were “very comfortable” performing skills increased from baseline for 27 of 34 skills post-training and 13 skills at six months. Half of respondents strongly agreed the course improved ability to manage COVID-19.

**Conclusions:**

This study demonstrates the feasibility of the Basic Emergency Care course to train emergency and inpatient healthcare workers with lasting impact. The timing of the study, at the beginning of the COVID-19 pandemic, provided an opportunity to illustrate the strategic overlap between building human resource capacity for long-term health systems strengthening and COVID-19. Future efforts should focus on integration with national training curricula and training of the trainers for broader dissemination and implementation at scale.

**Supplementary Information:**

The online version contains supplementary material available at 10.1186/s12913-022-07556-8.

## Introduction

The West African Ebola virus disease epidemic of 2014–2016 highlighted the vulnerability of the region’s chronically understaffed and underresourced health systems [1, 2]. One of the major barriers to controlling the epidemic in Sierra Leone, Guinea, and Liberia was lack of trained healthcare workers [2]. During the epidemic, the Sierra Leone Ministry of Health and Sanitation (MoHS) developed a long-term strategy for strengthening the national health system which included healthcare worker career development and capacity building as one of four pillars [1]. Today, global shortages of human resources for health present an ongoing challenge as hospitals around the world struggle to maintain essential services while also providing care for patients with severe and critical COVID-19.

Data from the Ebola virus disease epidemic demonstrated that providing critical care in resource-limited settings during disease outbreaks is feasible and can save lives [3]. With approximately 18.5% of COVID-19 cases progressing to severe or critical disease [4], the provision of emergency and critical care services is essential. Unfortunately, there is a significant outcome gap in Africa relative to the rest of the world, with excess mortality following intensive care unit admission for COVID-19 estimated between 11 and 23 deaths for every 100 patients [5]. Effective COVID-19 care depends in part on the ability to recognize severe illness and rapidly deliver life-saving interventions. The scarcity of skilled healthcare workers in many low-income countries (LICs), where emergency and critical care training is limited, poses a significant challenge for COVID-19 response, and is likely one of several factors contributing to outcome disparities.

Meeting the urgent need to identify methods for training multiple cadres of healthcare workers to manage COVID-19 patients has potential benefits that extend well beyond the pandemic. Training healthcare workers to recognize, assess, and stabilize emergency and critical conditions will not only help fight current and future epidemics but will also strengthen the long-term delivery of comprehensive healthcare in low- and-middle-income countries [6] where 54% of annual deaths are from conditions potentially treatable by prehospital and facility-based emergency care [7].

The World Health Organization (WHO) and International Committee of the Red Cross’s Basic Emergency Care Course (BEC) is an open-access five-day course for front-line emergency care providers in resource-limited settings [8] that has been implemented in multiple countries including Ethiopia [9], Zambia [10], Uganda [11], Tanzania [11], and Nigeria [12]. The BEC employs lectures and hands-on skill-based exercises to train providers in the assessment and management of emergency conditions [8]. Typically, BEC courses only enrol emergency unit staff, but the concepts and skills are relevant to inpatient management as well. The BEC course’s concepts could serve as a foundation both to cross-train personnel for COVID-19 care and to help maintain and strengthen essential health services in face of the pandemic. In early 2020, just prior to the start of the global pandemic we taught an expanded version of the BEC course in Sierra Leone, a LIC in West Africa, targeting healthcare workers from medical inpatient wards and intensive care units in addition to emergency units. To evaluate the impact of the course on healthcare workers’ knowledge and confidence in providing emergency and critical care services, we conducted a prospective cohort study of course participants, which coincided with the onset of the COVID-19 pandemic.

## Methods

### Setting

We conducted a prospective cohort study at two hospitals in Sierra Leone, a West African country of over 7.5 million people [13]. Ranked 182^nd^ of 189 countries by the Human Development Index [14] and with a life expectancy that is only 54.7 years [14], Sierra Leone is among the least developed countries worldwide. The 2014–2016 Ebola virus disease epidemic devastated an already fragile health system—staff losses and shaken community confidence in health facilities led to further deterioration of health services and outcomes [15]. The trainings were held at two hospitals in Sierra Leone–Koidu Government Hospital (KGH) and Connaught Hospital. KGH is a public secondary hospital with 170 beds located in Kono, a rural district in eastern Sierra Leone, with a catchment population of 506,100 as of the most recent 2015 census [13]. Connaught Hospital, located in the capital of Freetown, has 300 beds and serves as the national’s main public tertiary hospital. The course content and study design were finalized prior to December 2019, when the first reports of COVID-19 became public.

The Sierra Leone MoHS has emphasized that lack of human resources is “a major contributor to the poor health outcomes seen in Sierra Leone,” and identified “ongoing in-service training and support for all cadres” of healthcare workers as a key priority [16]. A nationwide shortage of doctors in Sierra Leone is exacerbated by their disproportionate concentration in urban areas. For example, Freetown has 1.1 doctors per 10,000 population [17] while Kono District has 0.08 doctors per 10,000 population [17]. In contrast, Community Health Officers—advanced practice providers with 3 years of training—are more evenly distributed across the country and are the principal frontline clinicians in many rural areas [17].

Emergency and critical care services are underdeveloped in Sierra Leone. An assessment of seven hospitals in Freetown found significant deficiencies in emergency and critical care services across multiple domains [18]. Only 67% of facilities had provided training to staff in adult emergency care and 50% had provided training for adult triage [18]. Sierra Leone does not have a formal postgraduate training program in emergency or critical care [18].

### Course structure

**Fig. 1:**
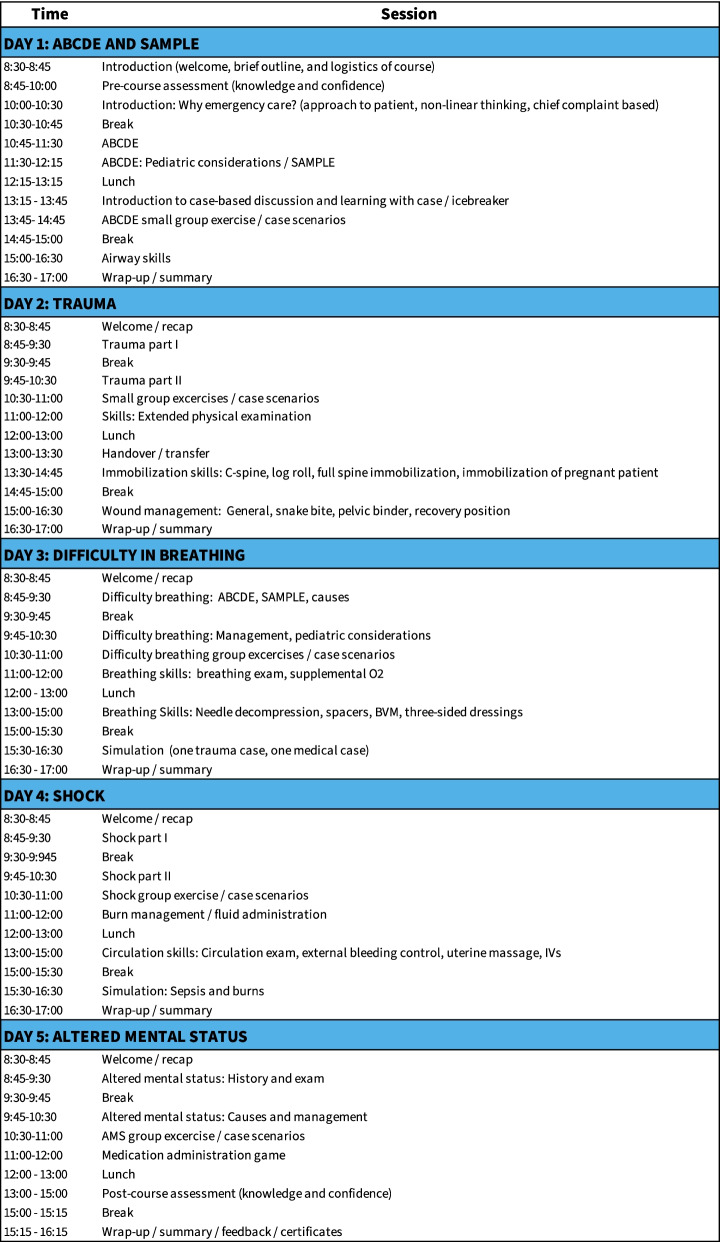
Course content and schedule

The BEC course is a five day, in-person training consisting of lectures and hands-on skills stations [8]. The course emphasizes a systematic method for assessment and stabilization of all patients using the ABCDE (airway, breathing, circulation, disability, and exposure) approach [8]. The first day is an introduction to the ABCDE approach with each of the subsequent four days focussed on applying this approach to specific conditions (Fig. 1). Although developed prior to the COVID-19 pandemic, much of the BEC course content has direct relevance to COVID-19. Notably, the third day is devoted to acute presentations of difficulty in breathing. Specific sessions include lectures on assessment, evaluation, and management; interactive case presentations; and hands-on teaching of essential skills such as basic airway manoeuvres, basic airway device insertion, oxygen administration, and bag-valve-mask ventilation. The training courses in Kono and Freetown were conducted sequentially during February 2020 in collaboration with the non-profit organization Partners In Health (PIH). Attendance and successful completion of all course components, demonstrated proficiency in all skill stations, and a score above 75% on the post-course knowledge assessment were required to pass the course. Four facilitators delivered the courses, with a trainer to trainee ratio of 1:3.8 and 1:4.5 for Kono and Freetown, respectively. The facilitators included two physicians from the United States and one clinician and one nurse from Sierra Leone. Facilitators were selected based on leadership ability, prior teaching experience, and emergency and critical care experience. Both facilitators from Sierra Leone had previously taken the BEC course. Content covered over the five days of the courses included all core BEC content.

### Participants

Nurses, community health officers, and doctors providing clinical care for critically ill patients in emergency or inpatient settings in Sierra Leone were eligible for invitation to the training. Participants were nominated by staff from PIH and MoHS. Eligibility was not restricted to Connaught Hospital or KGH—participants could come from any public sector hospital in Sierra Leone. All participants were over the age of 18. The Partners Institutional Review Board (Protocol#: 2020P000209) and Sierra Leone’s National Ethics and Scientific Review Committee provided ethical approval.

### Data collection

Pre- and immediate post-course assessments of knowledge and confidence are an essential component of the BEC course and were administered to all participants regardless of their participation in the study. On the first day of each training, all participants were approached for written informed consent and only data from those who provided consent were included in the study. Pre-training assessments were administered on the first day of each course and immediate post-training assessments were administered on the final day of each course. Both pre- and immediate post-training assessments were completed using paper forms. To assess knowledge retention and durability of impact, study participants were also asked to complete knowledge and confidence assessments at six months. Recognizing that the COVID-19 pandemic had created an increased demand for emergency and critical care services between initial study design and the pre-planned six month assessment, we developed a questionnaire with ten items related to COVID-19 and amended the study protocol to administer the COVID-19 questionnaire, in addition to the pre-planned knowledge and confidence assessments, at six months.

Data were managed using REDCap electronic data capture tools [19]. Results from completed paper versions of the pre- and immediate post-course knowledge and confidence assessments were manually entered into the REDCap database. The six month follow-up was conducted by sending participants links to online versions of the knowledge, confidence, and COVID-19 assessments via email and SMS/WhatsApp messages using the survey function in REDCap. Two weeks later, participants yet to complete the six month assessments were sent reminders via phone call and SMS/WhatsApp.

### Instruments

We assessed participant knowledge pre-course, immediately post-course, and at six months using a multiple-choice assessment containing 25 questions on course content which is a standardized component of the BEC course materials. We reported participant data as the percent of questions answered correctly.

Participant confidence was also assessed pre-course, immediately post-course, and at six months. Using a standardized instrument, participants rated their confidence performing 34 course skills divided into the domains of 1) overall confidence and skills and 2) specific skills. For each skill, participants rated their confidence on a Likert-scale of 1 (“not comfortable”) to 7 (“very comfortable”). For each skill, we reported the number and proportion of participants responding “very comfortable”. This outcome was selected for consistency with prior studies [10, 12, 20]. For the COVID-19 assessment, a group of content experts developed and refined ten questions on experience and comfort treating COVID-19. These questions were completed as part of the six month assessment. Four were “yes/no” questions, two asked participants to rate their agreement with statements on a Likert scale of 1 (“strongly disagree”) to 7 (“strongly agree”), and the remaining four asked participants to rate specific COVID-19 skills on a Likert-scale of 1 (“not comfortable”) to 7 (“very comfortable”). We report the number and proportion of participants responding “yes”, "strongly agree", or “very comfortable”.

### Statistical analysis

Data were analysed in Stata (Release 16). All participants that completed the course (i.e., attended all five days and completed immediate post-course knowledge and confidence assessments) were included in analyses. A passing score on the post-course knowledge assessment was defined as greater than 75% (at least 19 out of 25 questions) correct. Categorical variables were described using frequencies and proportions. Given the differences in training sites (urban tertiary vs. rural secondary) we also broke down outcome reporting by training site. Continuous variables were summarized by means, 95% confidence intervals, medians, and interquartile ranges. We compared mean test scores using paired Student’s t-tests and proportions of participants “very comfortable” with course skills using McNemar’s exact chi-squared tests for paired data. We used Wilcoxon matched-pairs signed-rank test for comparing the median number of skills participants rated “very comfortable”.

In additional analyses we compared mean knowledge scores by follow-up status using a two sided Student’s t-test. We also compared median number of skills participants rated “very comfortable” by follow-up status using Wilcoxon rank-sum test.

## Results

### Participant characteristics

**Fig. 2:**
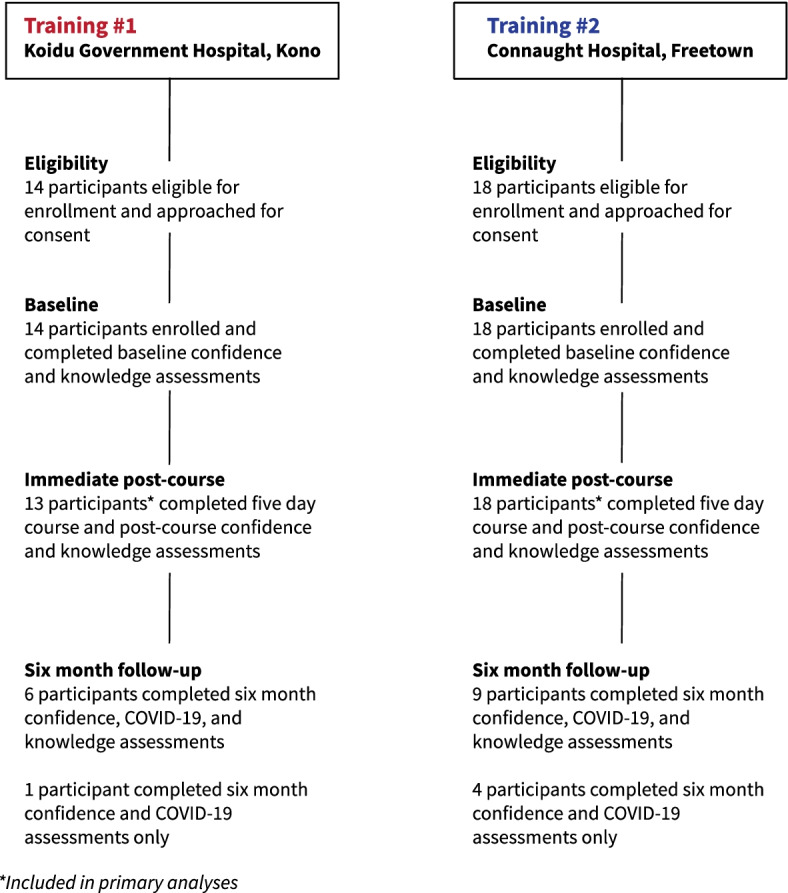
Participant enrolment

We enrolled all 32 eligible participants, 14 (44%) in Kono training and 18 (56%) in Freetown, all of whom attended the first day of the course and completed baseline assessments. All participants approached for consent agreed to participate in the study. An additional course participant joined after the pre-course assessments and was not eligible for enrolment. A total of 31 (97%) participants completed all five days of the course and immediate post-course knowledge and confidence assessments and were included in analyses (Fig. 2).

Most participants were nurses (84%), followed by community health officers (10%), and doctors (3%). Participants from the Kono training worked in the medical ward (54%), emergency unit (38%), and intensive care unit (8%). Participants from the Freetown training worked in the medical ward (61%), emergency unit (22%), and intensive care unit (17%). At six months, follow-up confidence assessment data—including additional COVID-19 questions—were collected from 20 (65%) participants and follow-up knowledge assessment data were collected from 15 (48%) participants (Table 1). The median time from completion of the course to follow-up was 187 days (IQR: 172 to 234).

**Table 1:** Participant characteristics

	**Kono** **(*n* = 13)**	**Freetown** **(*n* = 18)**	**Combined** **(*n* = 31)**
Completed immediate post-training confidence assessment n (%)	13 (100%)	18 (100%)	31 (100%)
Completed immediate post-training knowledge assessment n (%)	13 (100%)	18 (100%)	31 (100%)
Completed month 6 confidence assessment n (%)	7 (54%)	13 (72%)	20 (65%)
Completed month 6 knowledge assessment n (%)	6 (46%)	9 (50%)	15 (48%)
**Work area**			
Intensive care unit n (%)	1 (8%)	3 (17%)	4 (13%)
Emergency unit n (%)	5 (38%)	4 (22%)	5 (16%)
Medical ward n (%)	7 (54%)	11 (61%)	22 (71%)
**Role**			
Nurse n (%)	10 (77%)	16 (89%)	26 (84%)
Community health officer n (%)	3 (23%)	0	3 (10%)
Doctor n (%)	0	1 (6%)	1 (3%)
Other n (%)	0	1 (6%)	1 (3%)

### Knowledge assessment

**Fig. 3:**
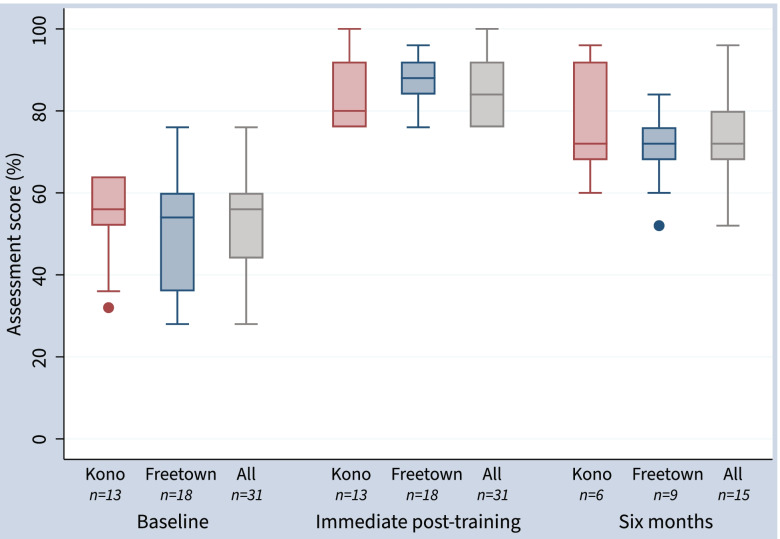
Knowledge assessment score over time by training group, Box plots of assessment scores by group and follow-up

**Fig. 4:**
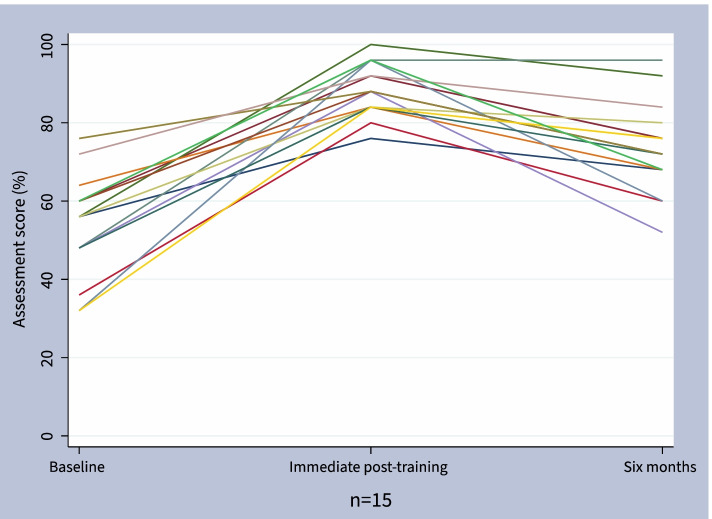
Individual knowledge assessment scores over time, Each line represents one of the 15 participants with complete data for baseline, immediate-post training, and six month knowledge assessments

Immediately post-training, there was a significant increase in knowledge assessment score among the Kono and Freetown groups compared to baseline (Table 2, Fig. 3) with all participants scoring above the required 75% to pass the course. The Kono group improved from a baseline mean score of 55% (95% CI: 48% to 61%) to 83% (78% to 89%, p < 0.001) immediate post-training. The Freetown cohort improved from a mean score of 51% (44% to 58%) at baseline to 86% (83% to 90%, *p* < 0.001) immediate post-training. Overall, participant scores increased by a mean of 33% (28% to 38%) from baseline to immediate post-training. Among the 15 participants who completed the six month follow-up knowledge assessment, there was a sustained mean improvement of 19% (11% to 28%, p-value 0.001) from baseline (Fig. 4). However, compared to the immediate post-course assessment there was a mean decrease of 15% (9% to 21%, *p*-value < 0.001). Compared to participants who were lost to follow-up, participants who completed six month follow-up had similar baseline knowledge assessment scores but higher post-course scores (Table S1).

**Table 2:** Knowledge assessment scores

	**Baseline**		**Post-training**			**Six months**		
	*n*	mean (95% CI)	*n*	mean (95% CI)	*p*-value*	*n*	mean (95% CI)	*p*-value^
Kono	13	55% (48% to 61%)	13	83% (78% to 89%)	< 0.001	6	77% (62% to 92%)	0.07
Freetown	18	51% (44% to 58%)	18	86% (83% to 90%)	< 0.001	9	71% (63% to 78%)	0.008
Combined	31	53% (48% to 57%)	31	85% (82% to 88%)	< 0.001	15	73% (67% to 80%)	0.001

^*^Two-sided paired t-test compared to baseline

^Two-sided paired t-test compared to baseline

### Confidence assessment

At baseline, individual participants reported feeling “very comfortable” performing a median of 9 (1 to 13) of the 34 skills covered in the course. Immediately post-training, the median number of “very comfortable” skills for individual participants increased to 25 (11 to 32, *p* < 0.001). This increase from baseline persisted at six months, with participants reporting a median of 21 (16 to 24) “very comfortable” skills (*p*-value < 0.001). The overall proportion of participants who felt “very comfortable” increased for 27 of the course skills immediately post-training (Tables 3 and S2). Significant improvements were seen in skills relevant to COVID-19 including assessment and management of patients with difficulty breathing, fever, chest pain, and altered mental status as well as skills related to management of airway and breathing pathology (Table 3). Initial increases in proportion of “very comfortable” participants were sustained at six month follow-up for 12 (44%) skills. In secondary analyses comparing proportion of participants with comfort ratings ≥ 6 (out of 7), increases were sustained at six month follow-up for 10 skills (Table S3). The percentage of participants feeling “very comfortable” with oxygen administration at baseline (61%) was significantly increased at six month follow-up (95%, *p*-value 0.03).

**Table 3:** Confidence assessment ratings of “very comfortable” for selected skills

	**Baseline** **(*n* = 31)**	**Post-training** **(*n* = 31)**		**Six months** **(*n* = 20)**	
	*n* (%)	*n* (%)	*p*-value*	*n* (%)	*p*-value^
Assessing patients in an emergency department	11 (35%)	19 (61%)	0.02	14 (70%)	0.11
Determining if a patient is stable or unstable	8 (26%)	16 (52%)	0.02	13 (65%)	0.03
Recognizing signs of shock	11 (35%)	19 (61%)	0.06	16 (80%)	0.07
Recognizing signs of sepsis	3 (10%)	18 (58%)	< 0.001	10 (50%)	0.008
Recognizing and assessing altered mental status	4 (13%)	18 (58%)	< 0.001	8 (40%)	0.02
Assessment and management of patient with difficulty breathing	8 (26%)	22 (71%)	< 0.001	17 (85%)	0.002
Assessment and management of patient with fever	14 (45%)	25 (81%)	0.005	18 (90%)	0.02
Suctioning the airway	13 (42%)	21 (68%)	0.06	16 (80%)	0.008
Repositioning the airway	8 (26%)	20 (65%)	0.004	14 (70%)	0.02
Inserting an oral airway	6 (19%)	19 (61%)	0.001	13 (65%)	0.04
Inserting a nasopharyngeal airway	6 (19%)	17 (55%)	0.007	11 (55%)	0.11
Managing a choking patient	2 (6%)	17 (55%)	< 0.001	8 (40%)	0.07
Administering oxygen	19 (61%)	22 (71%)	0.51	19 (95%)	0.03
Using a bag valve mask	9 (29%)	20 (65%)	0.01	13 (65%)	0.02
Performing a needle thoracostomy	2 (6%)	15 (48%)	0.001	1 (5%)	1.0

^*^McNemar’s exact chi-squared for paired data between baseline and immediate post-training

^McNemar’s exact chi-squared for paired data between baseline and month six

*AVPU* Alert, voice, pain, unresponsive, *ABCDE* Airway, breathing, circulation, disability, exposure

### COVID-19 assessment

At six months, 8 (40%) participants reported caring for patients with COVID-19 and 18 (90%) reported caring for patients with suspected COVID-19. Half the participants who completed the six month follow-up assessment and 63% of those who had cared for patients with confirmed COVID-19 strongly agreed that the course improved their ability to manage patients with COVID-19 (Table 4). Further, 60% of participants reported they were “very comfortable” recognizing signs of severe COVID-19 and 55% were “very comfortable” assessing respiratory status of patients with confirmed or suspected COVID-19. Among participants who had cared for patients with confirmed COVID-19, 75% were very comfortable with both of these skills.

**Table 4:** COVID-19 assessment at six months

	**Participants** **(*n* = 20)**
Provided direct clinical care for patients with confirmed COVID-19 n (%)	8 (40%)
Provided direct clinical care for patients with suspected COVID-19 n (%)	18 (90%)
**Participants reporting “strongly agree”**	
Have sufficient experience to manage patients with COVID-19 n (%)	9 (45%)
This course has improved ability to manage patients with COVID-19 n (%)	10 (50%)
**Participants reporting “very comfortable”**	
Caring for patients with COVID-19 n (%)	10 (50%)
Performing an initial assessment of patients with confirmed or suspected COVID-19 n (%)	11 (55%)
Recognizing signs of severe COVID-19 n (%)	12 (60%)
Assessing respiratory status of patients with confirmed or suspected COVID-19 n (%)	11 (55%)
Administering oxygen to patients with confirmed or suspected COVID-19 n (%)	10 (50%)
Assessing patients with confirmed or suspected COVID-19 for signs of shock n (%)	8 (40%)

## Discussion

This prospective cohort study assessing the impact of the BEC course at two hospitals in Sierra Leone early in the COVID-19 outbreak found improvements from baseline in participant knowledge and confidence in emergency and critical care skills immediately post-course and at six months, demonstrating the durability of the training’s impact. Notably, improvements in immediate post-course knowledge were similar in magnitude to prior BEC assessments [10–12] despite broadening inclusion criteria to staff from general medical wards and intensive care units, suggesting a role for this course beyond the emergency unit. To our knowledge, this is only the second study to conduct long-term follow-up of BEC training [21].

This study was conducted early in the COVID-19 outbreak—the WHO declared a pandemic 11 days after completion of the trainings. Although only 8 (40%) participants reported providing clinical care for patients with confirmed COVID-19, availability of COVID-19 diagnostics was very limited in Sierra Leone at the time of the six month follow-up. Therefore, the 18 (90%) participants who reported treating patients with suspected COVID-19 are likely a more accurate estimate of the true number treating COVID-19. Half of respondents at month six “strongly agreed” that the course improved ability to manage patients with COVID-19, and confidence in tasks related to respiratory care improved, such as using a bag valve mask and overall confidence managing patients with difficulty in breathing and fever. However, the improvement in oxygen delivery confidence at six months may not be attributable to the BEC course and suggests that efforts to provide oxygen delivery training in Sierra Leone during the COVID-19 pandemic were successful outside of this study. Though not the initial intent of our study, our results suggest that the BEC course, designed for long-term emergency health systems strengthening, could be used to prepare frontline healthcare workers for COVID-19 care. Indeed, OpenWHO has since developed COVID-19 training modules drawing on BEC course content [22]. COVID-19 has reinforced the need to shift to a systems-focused approach that spans medical specialties. We have demonstrated a practical example of how to begin to implement that shift in human resources for health by using the BEC course to train both emergency unit and inpatient staff from multiple facilities, although this may also be achievable with other courses. More broadly, these results highlight the potential to strengthen health system resilience through emergency and critical care training for frontline healthcare workers. Compared with the five prior assessments of the BEC course [10–12, 20, 21], participants’ baseline knowledge assessment scores were the among the lowest. However, the increase in knowledge assessment scores from baseline to immediate post-course was the highest recorded. The only study reporting knowledge assessment scores from longer term follow-up [21] reported an increase of 16% from baseline at seven months, which is similar to our observed increase of 19% at six months. Of note, we observed that most of the skills with reductions in confidence from immediate post-training to month six were in hands-on skills (log roll, fracture immobilization, needle thoracostomy). This suggests a potential future avenue of inquiry to better understand where refresher trainings would be most useful.

### Future directions

These findings highlight the importance of training interventions to improve emergency and critical care capacity in LICs, for COVID-19 as well as long-term health systems strengthening. Future efforts should be directed at integration with national training curricula for all cadres of front-line healthcare workers to facilitate broader dissemination and maximize impact. Particular attention should be paid to training of the trainers to expedite implementation at scale. Future investigations should explore the impact of providing refresher trainings with an emphasis of hands-on skills.

### Limitations

This study has several limitations. Sample size was limited to 31 participants from two locations in Sierra Leone and it is unclear whether the results would generalize to other LICs. Second, we assessed confidence in skills rather than measuring performance. Follow-up studies should examine patient management in the unit to evaluate if changes in care quality or outcomes occur. Third, there is potential for reporting bias if participants wanted to rate their confidence higher after the training course. However, participants’ knowledge assessment scores also improved, which is encouraging. Fourth, we did not track other potential trainings that participants may have received between the BEC course and the six month follow-up. Additional trainings in oxygen delivery in response to the COVID-19 pandemic could explain why participants’ confidence scores for administering oxygen were significantly higher at six months but not immediate post-training. It is unknown if other trainings could have affected six-month scores through recall bias. However, it is reassuring that the knowledge assessment results at six months are similar to previously published data [21].

Finally, the COVID-19 pandemic posed unique challenges to follow-up efforts. The original study design had called for in-person administration of instruments at six months. However, COVID-19 restrictions made this infeasible and the study protocol was revised to allow for the use of REDCap online survey tools. It is likely that using the online forms presented a barrier for some participants, either through mobile data costs or user interface. Indeed, there were five participants who completed the confidence instrument but did not proceed to the knowledge instrument. Although participants that were lost to follow-up at six months had lower post-course knowledge scores, the use of paired statistical tests allows reliable conclusions on the impact at six months for the 15 participants with follow-up data, if not for the entire group.

## Conclusions

This study demonstrates that the BEC course can be used to train both emergency and inpatient healthcare workers with lasting impact. Additionally, the timing of the course early in the COVID-19 pandemic provided an opportunity to illustrate the strategic overlap between building human resource capacity for long-term health systems strengthening and a respiratory pandemic. Future efforts should focus on integrating the BEC course with the national training curriculum and on training of the trainers to allow broader dissemination and implementation at scale.

## Supplementary Information

**Additional file 1.** Supplement to: Applying the WHO-ICRC BEC course to train emergency and inpatient healthcare workers in Sierra Leone early in the COVID-19 outbreak

## Data Availability

The datasets used and/or analysed during the current study are available from the corresponding author on reasonable request.

## Abbreviations

ABCDE: Airway, breathing, circulation, disability, and exposure
AVPU: Alert, voice, pain, unresponsive
BEC: Basic emergency care
KGH: Koidu Government Hospital
LIC: Low-income country
MoHS: Ministry of Health and Sanitation
PIH: Partners In Health
WHO: World Health Organization
