# Supplementary material for: Applying the WHO-ICRC BEC course to train emergency and inpatient healthcare workers in Sierra Leone early in the COVID-19 outbreak
Source: BMC Health Serv Res. 2022 Feb 14;22:197. doi: 10.1186/s12913-022-07556-8 (PMC8842917; doi:10.1186/s12913-022-07556-8)
Supplement: Supplementary file 1 — Additional file 1. Supplement to: Applying the WHO-ICRC BEC course to train emergency and inpatient healthcare workers in Sierra Leone early in the COVID-19 outbreak [file 12913_2022_7556_MOESM1_ESM.docx]

**Supplement to:** Applying the WHO-ICRC BEC course to train emergency and inpatient healthcare workers in Sierra Leone early in the COVID-19 outbreak

*Paul D. Sonenthal MD^1,2,3^, Chiyembekezo Kachimanga MBBS MPhil^4^, Doris Komba RN^5^, Moses Bangura CHO^4^, Nicholas Ludmer MD^6^, Marta Lado MD DTMH^4^, Marta Patino MD DTMH^4^, Rachel B. Gerrard MPhil*^3^*, Matthew J. Vandy MD MSc^5^, Regan H. Marsh MD MPH^2,3,7^, Joia Mukherjee MD MPH^2,3^, Shada A. Rouhani MD MPH^2,3,7^*

[Table S1. Baseline and immediate post-course outcomes by follow-up status 1](#_Toc94028192)

[Table S2. Confidence assessment ratings of “very comfortable” for additional skills 2](#_Toc94028193)

[Table S3. Confidence assessment ratings of >6 out of 7 3](#_Toc94028194)

| Table S1. Baseline and immediate post-course outcomes by follow-up status | | | |
| --- | --- | --- | --- |
|  | **Lost to follow-up at six months** | **Completed six month follow-up** | *p-value* |
| ***Knowledge score*** |  |  |  |
| Participants *n* | 16 | 15 |  |
| Baseline *mean (95% CI)* | 52% (45% to 58%) | 54% (46% to 61%) | 0.65^ |
| Immediate post-course *mean (95% CI)* | 82% (78% to 86%) | 89% (85% to 92%) | 0.02^ |
| ***“Very comfortable” skills*** |  |  |  |
| Participants *n* | 11 | 20 |  |
| Baseline *median (IQR)* | 11 (1 to 13) | 9 (2 to 14) | 0.99* |
| Immediate post-course *median (IQR)* | 24 (7 to 32) | 28 (16 to 32) | 0.44* |
| ^p-value for two sided Student’s t-test  *p-value for Wilcoxon rank-sum test | | | |

| Table S2. Confidence assessment ratings of “very comfortable” for additional skills | | | | | |
| --- | --- | --- | --- | --- | --- |
|  | **Baseline**  **(n=31)** | **Post-training**  **(n=31)** | | **Six months**  **(n=20)** | |
|  | *n (%)* | *n (%)* | *p-value** | *n (%)* | *p-value^* |
| **Comfort with assessment and management of:** |  |  |  |  |  |
| Patient with choking | 2 (6%) | 22 (71%) | <0.001 | 9 (45%) | 0.07 |
| Patient with altered mental status | 1 (3%) | 20 (65%) | <0.001 | 5 (25%) | 0.06 |
| Patient with chest pain | 6 (19%) | 19 (61%) | 0.004 | 8 (40%) | 0.07 |
| Patient with abdominal pain | 8 (26%) | 20 (65%) | 0.004 | 11 (55%) | 0.07 |
| Patient who presents following a trauma | 2 (6%) | 24 (77%) | <0.001 | 8 (40%) | 0.016 |
| **Comfort with the following skills:** |  |  |  |  |  |
| ***Circulation skills*** |  |  |  |  |  |
| Applying pressure to a wound | 14 (45%) | 22 (71%) | 0.008 | 15 (75%) | 0.07 |
| Applying a tourniquet | 13 (42%) | 19 (61%) | 0.15 | 16 (80%) | 0.18 |
| Administering fluids | 18 (58%) | 24 (77%) | 0.11 | 16 (80%) | 0.03 |
| ***Additional skills*** |  |  |  |  |  |
| Using the Glasgow Coma Scale | 8 (26%) | 14 (45%) | 0.15 | 10 (50%) | 0.06 |
| Using the AVPU scale | 13 (42%) | 23 (74%) | 0.01 | 16 (80%) | 0.008 |
| ***Trauma skills*** |  |  |  |  |  |
| Performing a primary survey (ABCDEs) | 7 (23%) | 21 (68%) | <0.001 | 13 (65%) | 0.02 |
| Performing a secondary survey | 1 (3%) | 17 (55%) | <0.001 | 7 (35%) | 0.07 |
| Stabilizing a patient’s cervical spine | 6 (19%) | 21 (68%) | <0.001 | 10 (50%) | 0.07 |
| Performing a log roll | 6 (19%) | 20 (65%) | <0.001 | 10 (50%) | 0.18 |
| Applying a pelvic binder | 1 (3%) | 22 (71%) | <0.001 | 8 (40%) | 0.04 |
| Fracture immobilization and splinting | 7 (23%) | 22 (71%) | <0.001 | 9 (45%) | 0.29 |
| ***Wound management*** |  |  |  |  |  |
| Incision and drainage of abscess | 10 (32%) | 12 (39%) | 0.79 | 6 (30%) | 1.0 |
| Burn management | 9 (29%) | 20 (65%) | 0.007 | 9 (45%) | 0.18 |
| Bite wound management | 7 (23%) | 22 (71%) | 0.002 | 7 (35%) | 0.13 |
| *McNemar’s exact chi-squared for paired data between baseline and immediate post-training  ^McNemar’s exact chi-squared for paired data between baseline and month six | | | | | |
| AVPU: Alert, voice, pain, unresponsive; ABCDE: Airway, breathing, circulation, disability, exposure | | | | | |

| Table S3. Confidence assessment ratings of >6 out of 7 | | | | | |
| --- | --- | --- | --- | --- | --- |
|  | **Baseline**  **(n=31)** | **Post-training**  **(n=31)** | | **Six months**  **(n=20)** | |
|  | *n (%)* | *n (%)* | *p-value** | *n (%)* | *p-value^* |
| Comfort assessing patients in an emergency department | 14 (45%) | 28 (90%) | <0.001 | 16 (80%) | 0.11 |
| Comfort determining if a patient is stable or unstable | 12 (39%) | 23 (74%) | 0.003 | 15 (75%) | 0.07 |
| Comfort recognizing signs of shock | 15 (48%) | 28 (90%) | <0.001 | 18 (90%) | 0.13 |
| Comfort recognizing signs of sepsis | 10 (32%) | 27 (87%) | <0.001 | 15 (75%) | 0.04 |
| Comfort recognizing and assessing altered mental status | 8 (26%) | 26 (84%) | <0.001 | 12 (60%) | 0.002 |
| **Comfort with assessment and management of:** |  |  |  |  |  |
| Patient with choking | 7 (23%) | 27 (87%) | <0.001 | 12 (60%) | 0.07 |
| Patient with difficulty breathing | 18 (58%) | 28 (90%) | 0.006 | 19 (95%) | 0.02 |
| Patient with altered mental status | 4 (13%) | 27 (87%) | <0.001 | 9 (45%) | 0.008 |
| Patient with chest pain | 10 (32%) | 27 (87%) | <0.001 | 12 (60%) | 0.11 |
| Patient with abdominal pain | 16 (52%) | 29 (94%) | <0.001 | 15 (75%) | 0.15 |
| Patient with fever | 21 (68%) | 29 (94%) | 0.008 | 19 (95%) | 0.07 |
| Patient who presents following a trauma | 10 (32%) | 28 (90%) | <0.001 | 10 (50%) | 0.45 |
| **Comfort with the following skills:** |  |  |  |  |  |
| ***Airway skills*** |  |  |  |  |  |
| Suctioning the airway | 19 (61%) | 27 (87%) | 0.02 | 18 (90%) | 0.06 |
| Repositioning the airway | 17 (55%) | 27 (87%) | 0.002 | 16 (80%) | 0.45 |
| Inserting an oral airway | 16 (52%) | 28 (90%) | <0.001 | 17 (85%) | 0.06 |
| Inserting a nasopharyngeal airway | 15 (48%) | 27 (87%) | <0.001 | 13 (65%) | 1.0 |
| Managing a choking patient | 12 (39%) | 25 (81%) | <0.001 | 16 (80%) | 0.04 |
| ***Breathing skills*** |  |  |  |  |  |
| Administering oxygen | 25 (81%) | 30 (97%) | 0.06 | 18 (90%) | 1.0 |
| Using a bag valve mask | 15 (48%) | 30 (97%) | <0.001 | 16 (80%) | 0.13 |
| Performing a needle thoracostomy | 3 (10%) | 23 (74%) | <0.001 | 5 (25%) | 0.22 |
| ***Circulation skills*** |  |  |  |  |  |
| Applying pressure to a wound | 20 (65%) | 29 (94%) | 0.004 | 20 (100%) | 0.02 |
| Applying a tourniquet | 17 (55%) | 26 (84%) | 0.02 | 19 (95%) | 0.13 |
| Administering fluids | 25 (81%) | 29 (94%) | 0.22 | 20 (100%) | 0.13 |
| ***Additional skills*** |  |  |  |  |  |
| Using the Glasgow Coma Scale | 13 (42%) | 24 (77%) | 0.01 | 13 (65%) | 0.13 |
| Using the AVPU scale | 19 (61%) | 29 (94%) | 0.006 | 17 (85%) | 0.06 |
| ***Trauma skills*** |  |  |  |  |  |
| Performing a primary survey (ABCDEs) | 13 (42%) | 29 (94%) | <0.001 | 14 (70%) | 0.18 |
| Performing a secondary survey | 8 (26%) | 27 (87%) | <0.001 | 12 (60%) | 0.008 |
| Stabilizing a patient’s cervical spine | 12 (39%) | 27 (87%) | <0.001 | 14 (70%) | 0.04 |
| Performing a log roll | 8 (26%) | 30 (97%) | <0.001 | 14 (70%) | 0.008 |
| Applying a pelvic binder | 4 (13%) | 28 (90%) | <0.001 | 12 (60%) | 0.002 |
| Fracture immobilization and splinting | 16 (52%) | 27 (87%) | <0.001 | 13 (65%) | 0.22 |
| ***Wound management*** |  |  |  |  |  |
| Incision and drainage of abscess | 15 (48%) | 19 (61%) | 0.34 | 10 (50%) | 1.0 |
| Burn management | 17 (55%) | 28 (90%) | 0.001 | 13 (65%) | 0.63 |
| Bite wound management | 17 (55%) | 29 (94%) | <0.001 | 13 (65%) | 0.69 |
| *McNemar’s exact chi-squared for paired data between baseline and immediate post-training  ^McNemar’s exact chi-squared for paired data between baseline and month six | | | | | |
| AVPU: Alert, voice, pain, unresponsive; ABCDE: Airway, breathing, circulation, disability, exposure | | | | | |
